# Genetic liability to sedentary behaviour and cardiovascular disease incidence in the FinnGen and HUNT cohorts

**DOI:** 10.1136/bjsports-2024-109491

**Published:** 2025-03-26

**Authors:** Laura Joensuu, Kaisa Koivunen, Niko Paavo Tynkkynen, Teemu Palviainen, Jaakko Kaprio, FinnGen Consortium, Marie Klevjer, Karsten Øvretveit, Ulrik Wisløff, Anja Bye, Ulf Ekelund, Elina Sillanpää

**Affiliations:** 1 Gerontology Research Center, Faculty of Sport and Health Sciences, University of Jyväskylä, Jyväskylä, Finland; 2 Institute for Molecular Medicine Finland, HiLIFE, University of Helsinki, Helsinki, Finland; 3 Cardiac Exercise Research Group, Department of Circulation and Medical Imaging, Faculty of Medicine and Health Sciences, Norwegian University of Science and Technology, Trondheim, Norway; 4 Clinic of Cardiology, St. Olav’s Hospital, Trondheim, Norway; 5 HUNT Center for Molecular and Clinical Epidemiology, Department of Public Health and Nursing, Norwegian University of Science and Technology, Trondheim, Norway; 6 Department of Sports Medicine, Norwegian School of Sport Sciences, Oslo, Norway; 7 Department for Chronic Diseases, Norwegian Institute of Public Health, Oslo, Norway; 8 Faculty of Sport and Health Sciences, University of Jyväskylä, Jyväskylä, Finland; 9 Wellbeing Services County of Central Finland, Jyväskylä, Finland

**Keywords:** Cardiology, Epidemiology, Genetics, Public health, Sports medicine

## Abstract

**Objective:**

Energy-saving sedentary behaviour may be an evolutionarily selected trait that is no longer advantageous. We investigated the associations between genetic liability to sedentary behaviour and the incidence of the most common cardiovascular disease (CVD).

**Methods:**

We constructed and validated a genome-wide polygenic score for leisure screen time (PGS LST) as a measure of genetic liability to sedentary behaviour. We performed survival analyses between higher PGS LST and register-based CVDs using the FinnGen cohort (N=293 250–333 012). Replication and exploratory analyses were conducted in an independent Norwegian Trøndelag Health Study (HUNT) cohort (N=35 289).

**Results:**

In FinnGen, each SD increase in PGS LST was associated with a higher risk of incident CVD (HR: 1.05 (95% CI 1.05 to 1.06)) (168 770 cases over 17 101 133 person-years). The magnitudes of association for the three most common CVDs were 1.09 ((95% CI 1.08 to 1.09), 1.06 ((95% CI 1.05 to 1.07) and 1.05 ((95% CI 1.04 to 1.06) for hypertensive disease, ischaemic heart disease and cerebrovascular disease, respectively. Those in the top decile of PGS LST had 21%, 35%, 26% and 19% higher risk of any CVD, hypertensive disease, ischaemic heart disease and cerebrovascular disease, respectively, than those in the bottom decile. Associations were replicated in HUNT and remained independent of covariates (socioeconomic status, body mass index and smoking) except for cerebrovascular disease. Besides direct effects, reduced physical activity served as a potential mediating pathway for the observed associations.

**Conclusions:**

We found that genetic liability to sedentary behaviour is associated with incident CVD, although effect sizes with current PGS remained small. These findings suggest that genetic liability to sedentary behaviour is an under-recognised driver of common CVDs.

**WHAT IS ALREADY KNOWN ON THIS TOPIC**

- Genetics are known to be associated with sedentary behaviour and incident cardiovascular disease, but it is not known whether these two phenotypes are driven by the same genotype.

**WHAT THIS STUDY ADDS**

- We observed that a genetic liability to sedentary behaviour mutually increased the risk of being sedentary and having a higher risk of common cardiovascular diseases in two different populations.

**HOW THIS STUDY MIGHT AFFECT RESEARCH, PRACTICE OR POLICY**

- This study provides novel insights into the relationship between genetic predisposition to sedentary behaviour and the development of cardiovascular disease, shedding light on a previously underexplored aspect of disease aetiology.
- Understanding the contribution of genetics to sedentary behaviour and related health consequences may motivate health professionals to develop novel physical activity interventions.

## Introduction

 Cardiovascular diseases (CVDs) are the leading cause of disease burden and death worldwide.^1^ Sedentary behaviour is any waking behaviour in a sitting, reclining or lying posture and characterised by an energy expenditure of less than or equal to 1.5 metabolic equivalents (METs).^2^ Adults spend up to 60% of their awake time sedentary.^3^ The prevalence of sedentary behaviours has remained stable or even increased during the last 20 years.^4^ Sedentary behaviours are systematically associated with increased risk of CVD incidence and related mortality in prospective observational cohort studies.^5 6^

Interestingly, energy-saving sedentary behaviour has been suggested to be a trait selected by evolution to ensure reproduction and survival.^7^ Thus, genetics may be an important driver of sedentary behaviour, as supported by previous evidence from twin and candidate gene studies.^8^ However, the potential effects of this genetic liability on morbidity are unclear. From an evolutionary perspective, it is plausible that genetic predisposition to sedentary behaviour increases the risk of common non-communicable diseases.^9 10^ It has been suggested that the rapid environmental changes in societies, particularly in Western countries, may have led to an ‘evolutionary mismatch’.^9^ In this scenario, the previously advantageous alleles, for example, the genetic liability for sedentary behaviour, may cause morbidity in the current postindustrial environment.^11^ Furthermore, as life expectancy has increased, the later life deleterious effects of underlying genetics may become more apparent.^10^ The theory of antagonistic pleiotropy suggests that some genes that have favoured survival during reproductive ages in the past may currently limit lifespan and increase morbidity.^12^ Underlying genetics may, therefore, be an important confounding factor in the commonly observed associations between sedentary behaviour and incident CVD.

Polygenic scores (PGSs) summarise the genome-wide information between single-nucleotide polymorphisms (SNPs) and a phenotype into a single variable that quantifies a person’s genetic liability for a given trait.^13^ In this study, we assess whether a higher genetic liability for sedentary behaviour is associated with an increased risk of incident CVD (based on individual-level data and measured by hazard ratios and incidence rates). Leisure screen time (LST) is a commonly reported voluntary mode of sedentary behaviour and thus serves as a proxy for this trait.^6^ We hypothesised that higher genetic liability for LST, as measured by a PGS LST, would be associated with an increased risk of developing CVD.

## Methods

**Figure 1:**
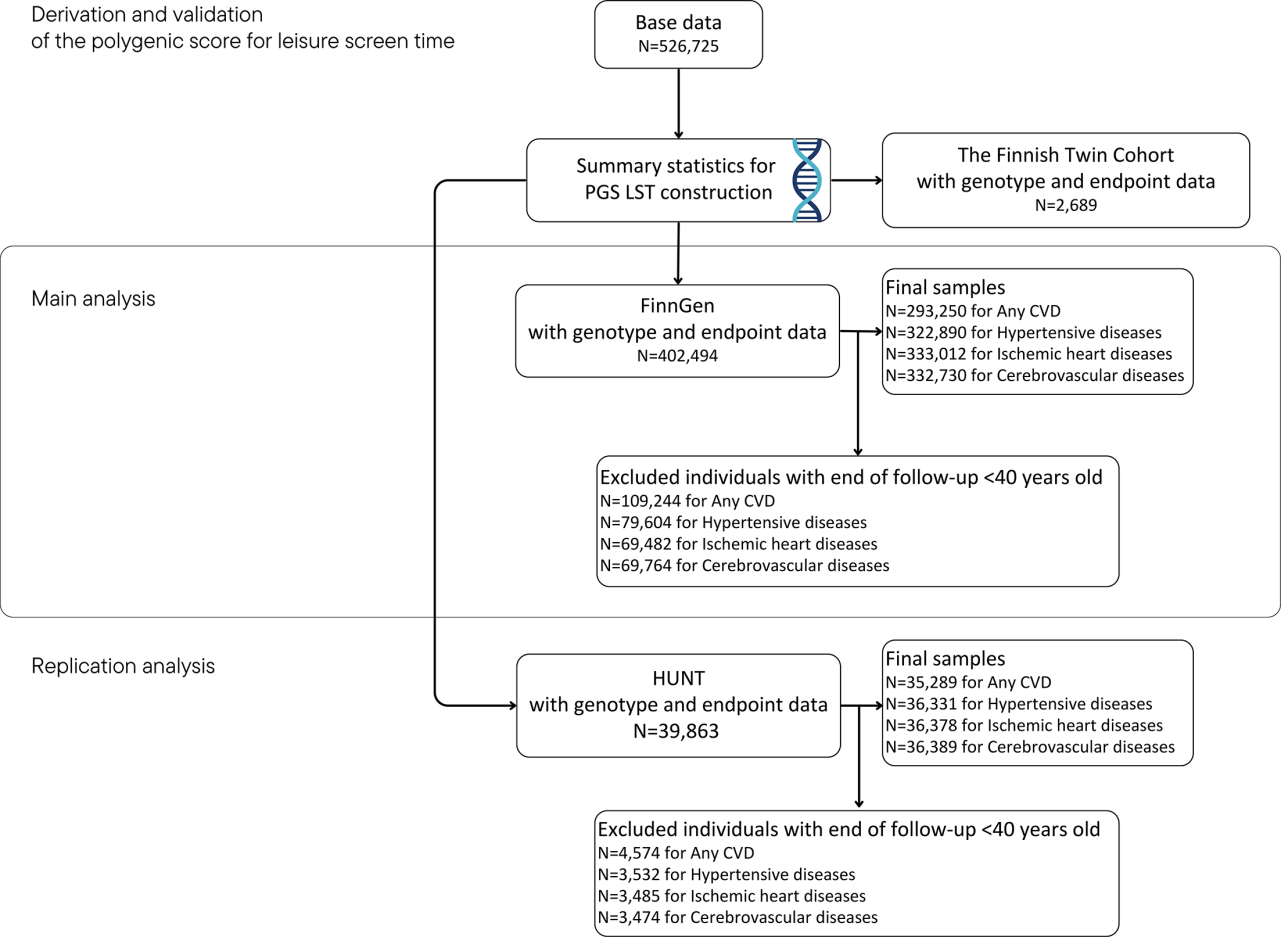
Study design and cohorts. CVD, cardiovascular disease; HUNT, Norwegian Trøndelag Health Study; PGS LST, polygenic score for leisure screen time.

This study consists of four phases. First, we constructed a PGS for LST. Second, we assessed the validity of this score against self-reported LST in the older Finnish Twin Cohort (FTC). Third, we assessed the associations between the PGS LST and incident CVD in the FinnGen cohort (~10% of the Finnish population). Finally, we replicated the analyses in an independent cohort (The Norwegian Trøndelag Health Study (the HUNT study)). The study design and cohorts are shown in figure 1. Additionally, we conducted exploratory analyses to estimate the potential explanations for the observed associations. Detailed information regarding the cohorts is available in the online supplemental methods.

### Outcomes

#### Leisure screen time

For the validation analyses in the FTC, LST in hours per day (hour/day) was used as the outcome and included self-reported television (TV) and video viewing, and computer use at home. This outcome was selected to match exactly the outcome used in the base data for PGS LST. In the FTC, participants reported how many hours per day they spent sitting: at home watching TV or videos, and at home at the computer using two separate questions. Both questions had four choices: (a) less than an hour, (b) an hour–less than 2 hours, (c) 2 hours–less than 4 hours and (d) 4 hours or more.^14^ Answers were summarised to indicate LST (hour/day). The data were collected in 2011 and represent screen time activities at that time.

#### Cardiovascular disease

Any CVD and the three most common CVDs in the FinnGen, that is, hypertensive, ischaemic heart and cerebrovascular diseases were selected. The CVD categorisation was based on FinnGen endpoint definitions and ICD-10 and ICD-9 codes detailed in the online supplemental methods. We used the same ICD codes in the HUNT replication analyses.

### Exposure

The PGS LST was based on the largest available genome-wide association study (GWAS) meta-analysis for LST at the time of the analysis (GWAS Catalogue Study ID: GCST90104339).^15^ The SNP heritability was 7.4% assessed by Complex-Traits Genetics Virtual Lab.^16^ The base data did not overlap with the later used FTC, FinnGen or HUNT study cohorts. The descriptives of the base data are described in online supplemental table 1.

We used the high-performing SBayesR approach to construct the PGS.^17^ In SBayesR, the base data summary statistics are reweighted to consider the linkage disequilibrium (LD) between each variant and restricted to HapMap3 SNPs to ensure computational efficiency while representing the whole genome. The SBayersR method does not use any specific p value threshold nor pruning for selecting the informative variants. The method uses Bayesian multiple regression models, where GWAS SNP effect estimates are scaled using a mixture of four alternative genetic effort distributions as the prior.^17 18^ The correlations between genetic variants (LD) were taken into account by using an external LD-reference panel generated by the SBayesR authors, constructed from a random sample of 50 000 UK Biobank individuals. These reweighted effect sizes (shown in online supplemental file 2) are then summarised to form a PGS, which indicates the genome-wide genetic liability for the trait of interest. The pipeline and relevant codes are available elsewhere.^19^ The final number of processed variants was 890 575 in the FTC, 891 628 in the FinnGen cohort and 901 640 in the HUNT cohort. We used standardised PGS LST scores (mean of 0, SD of 1) in the analyses.

#### Genotyping, quality control and imputation

Details related to genotyping procedures in the different cohorts are available in the online supplemental methods.

### Covariates

Covariates were based on previous literature.^20 21^ The first 10 principal components of ancestry adjust for any genetic stratification that may occur in the study population and were used alongside sex as a covariate in all analyses.^20^ Additional covariates were specific to the analysis. In validation analyses in the FTC, we also used age, educational attainment (highest achieved self-reported educational degree in the Finnish educational system; low, basic education degree at most; middle, basic education and additional studies; and high, at least upper secondary degree),^22^ and body mass index (BMI) based on valid self-reported height and weight.^23^ In main and replication analyses, we also used genotyping batch, which accounts for the potential stratification that may occur from the genotyping array and sample. In further exploratory analyses with the replication cohort, we controlled for variance caused by highly plausible covariates, such as BMI (based on measured height and weight (Jenix DS-102, Dong Sahn Jenix Co, Ltd, Korea)), socioeconomic status (SES; derived from occupational status)^21^ and self-reported smoking status (never, former, current) using the HUNT3 data.

### Statistical analysis

#### Validation analyses

All validation analyses were performed with Stata BE 18.0 (StataCorp). Correlation between PGS LST and self-reported LST was tested with Pearson correlation. The association between PGS LST and self-reported LST was assessed with linear regression in the FTC, where the twin structure of the data was acknowledged using the svyset command (ie, the twin pair was the primary sampling unit). The crude model included standardised PGS LST and the first ten principal components of ancestry. Model 1 was adjusted additionally for age and sex, and model 2 was further adjusted with educational attainment and BMI. Differences between high and low genetic liabilities were evaluated using linear regression, where a binary variable indicated whether the individual belonged to the highest or lowest decile of PGS LST. A priori power calculations indicated that sufficient statistical power would be reached with a sample size of N>950 (expected small effect size 0.02, 15 predictors, power at 0.8 level and α set to 0.05).

#### Main analyses

We used Cox proportional hazard models and R packages survival^24^ and survminer^25^ to estimate the hazard ratios (HRs) and 95% confidence intervals (CI) between PGS LST and incident CVD in the FinnGen cohort. Visual inspection of Schoenfeld residuals and log-minus-log plots indicated that the fully adjusted models satisfy the proportional hazard assumptions.^26^ PGS×SEX interaction was tested, but no replicable sex differences were observed; thus, analyses were not disaggregated by sex. We used age as the time scale and the first 10 principal components of ancestry, genotyping batch, and sex as covariates. The start of follow-up was set at birth, as the PGS remains stable from conception. The follow-up ended with whichever came first among the first record of the endpoint of interest, death or the end of follow-up on 31 December 2021. Using the weights of the European standard population, age-standardised incidence was calculated when relevant.^27^ We also compared the HRs (relative measures of survival) and cohort-specific cumulative incidence of events (absolute measures of survival) between the highest and lowest deciles of PGS LST. We additionally conducted competing risk analysis with Fine-Gray subdistribution hazard models for the three main CVDs, as one event may preclude the occurrence of another.^28^ Additionally, as the exposure (PGS LST) might affect who survives until genotyping and also to control for immortal time bias, we conducted sensitivity analyses in which the start of the follow-up was set to the date of individual DNA sampling. Sufficient statistical power was expected to be achieved with >120 cases, using a classical definition of 10 cases per predictor variable.

#### Replication analyses

The replication analyses were conducted in HUNT and followed a similar procedure as the main survival analyses, except that when mortality data were not available, the end of follow-up was assigned to whichever came first between the first record of the endpoint of interest and last verified contact with healthcare.

#### Exploratory analyses

We tested if the associations were independent of highly plausible covariates with a complete case subsample of HUNT3. In this analysis, model 1 included PGS LST, the first 10 principal components of ancestry, sex, and genotyping batch while model 2 included additionally SES, model 3 additionally BMI and model 4 additionally smoking status. We also assessed if adding PGSs for CVDs and physical activity (PA) (online supplemental methods) attenuates the main observations. We additionally tested a potential mediating pathway from PGS LST to incident CVD via sedentary behaviour using the product of coefficients approach and accompanying Sobel test, which are suitable for survival analysis with common outcomes.^29^ In this mediation analysis, we used participants from the sensitivity analyses with self-reported total sitting time (hour/day): “About how many hours do you sit during an average day? (include work hours and leisure time)”. Only diseases with an established association between PGS LST and incident disease were included in the mediation analysis. The association between PGS LST and total sitting time was evaluated with linear regression, while a Cox proportional hazards model was used for the association between total sitting time and disease incidence. The models were adjusted for sex similar to prior sensitivity analyses but not for the first ten principal components or genotyping batch for consistency across pathways. These mediating analyses were replicated with a PA variable, that is, MET hours per week, calculated as the product of self-reported exercise frequency, intensity and duration as previously described elsewhere.^21 30^ Across all analyses, all tests were two-tailed, and α was set at p<0.05.

#### Patient and public involvement

The participants were not involved in forming the research questions, outcome measures, design or other aspects of the study.

#### Equity, diversity and inclusion statement

The author group is gender-balanced, consisting of five women and six men. The team comprises PhD students, mid-career as well as senior researchers from diverse disciplines. The authors are from two countries. However, both represent high-income countries. Our study population, sampled on a population basis, includes both male and female subjects (52% women) from diverse socioeconomic backgrounds. However, those with access to healthcare might be over-represented in the sample.

## Results

### Association between PGS for LST and self-reported LST

The 2689 individuals of the FTC reported an average of 3.9±1.1 hours LST per day (online supplemental table 2). The effect size for PGS LST against LST was small (Pearson correlation coefficient=0.08, p<0.001), and PGS LST explained 0.7% of the variance in the LST. PGS LST of 1 SD higher was associated with higher self-reported LST (hour/day), with β=0.09 (95% CI 0.05 to 0.14) in the crude model; and β=0.08 (95% CI 0.04 to 0.13) in the fully adjusted model (online supplemental table 3). Correspondingly, individuals in the highest decile for PGS LST reported an average of 4.1±1.0 hour/day LST, while those in the lowest decile reported an average of 3.7 (1.1) hour/day (p<0.001 for difference) (online supplemental table 4).

### Association between PGS for LST and CVD incidence

Descriptive data on 293 250 participants in the FinnGen and 35 289 participants in the HUNT with any CVD are shown in table 1. The average follow-up time from birth was 58 (40 to 106) years (total of 17 101 133 person-years) in FinnGen and 64 (40 to 103) years (total of 2 257 493 person-years) in the HUNT.

**Table 1:** Descriptives of individuals with any CVD in the main FinnGen cohort and in the replication cohort (HUNT)

Characteristic	Main cohort		Replication cohort	
	N	FinnGen	N	HUNT
Age (years)	293 250	67.0 (13.0)	35 289	64.0 (13.1)
Female (%)	293 250	52.3	18 218	51.6
BMI (kg/m^2^)	82 224	27.8 (5.3)	22 398	27.9 (4.5)
Smoking status	48 391		21 768	
Never (%)	17 915	37.0	7921	36.4
Former (%)	7248	15.0	8607	39.5
Current (%)	23 228	48.0	5240	24.1

Values are presented as means (SD) for continuous variables and percentages for others. Age is from the end of follow-up, and others the last known information during adulthood; FinnGen data are from FinnGen data release 11.

BMI, body mass index; CVD, cardiovascular disease; HUNT, Norwegian Trøndelag Health Study.

**Figure 2:**
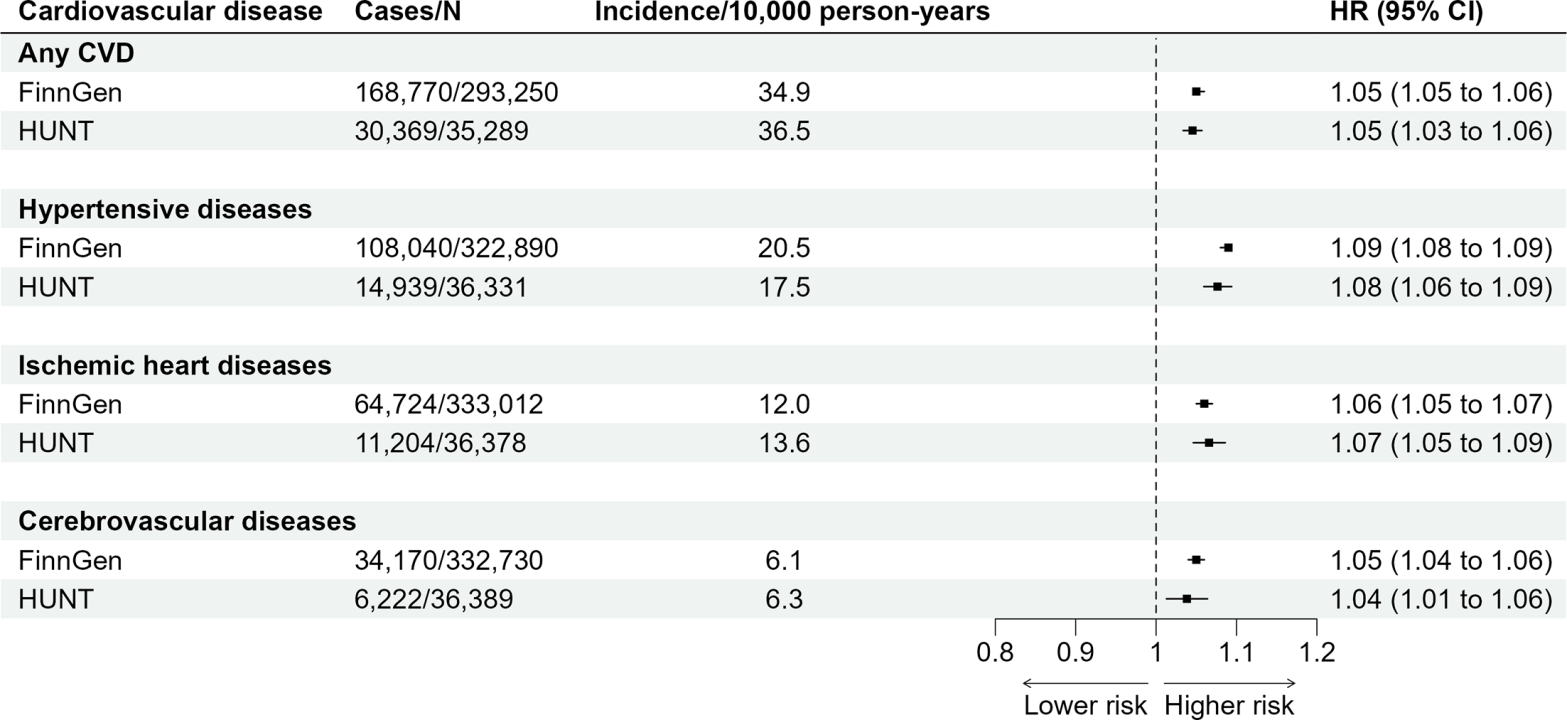
Associations between polygenic score for leisure screen time and CVD incidence. Values are HRs and 95% CIs per 1 SD increase in the polygenic score for leisure screen time. We used age as the time scale, and the first 10 principal components of ancestry, sex and genotyping batch as covariates. Incidence values are age-standardised according to the European Standard Population to enable comparison between FinnGen and HUNT cohorts. CVD, cardiovascular disease; HUNT, Norwegian Trøndelag Health Study.

In FinnGen, PGS LST of 1 SD higher was associated with a higher risk of any CVD after adjusting for the first 10 principal components of ancestry, sex and genotyping batch (HR 1.05, 95% CI 1.05 to 1.06; figure 2). Disease-specific associations for the three most common CVDs were (1) 1.09 (95% CI 1.08 to 1.09) for hypertensive diseases (108 040 cases), (2) 1.06 (95% CI 1.05 to 1.07) for ischaemic heart diseases (64 724 cases) and (3) 1.05 (95% CI 1.04 to 1.06) for cerebrovascular diseases (34 170 cases). The associations were replicated in the HUNT cohort with exactly or very similar effect sizes (figure 2).

**Figure 3:**
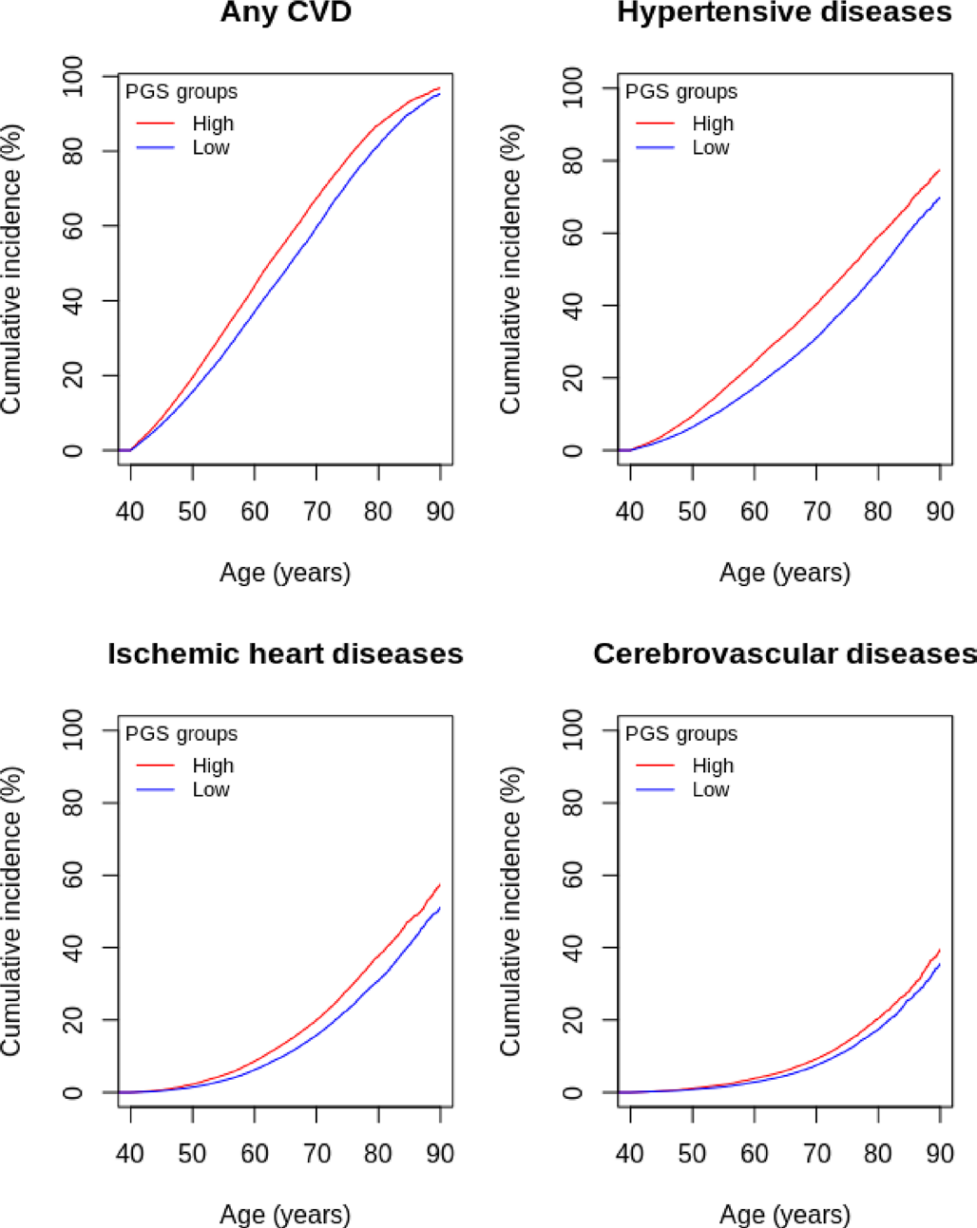
Cumulative incidence curves for high (>90th percentile (red)) and low (<10th percentile (blue)) PGS LST groups in the FinnGen cohort based on Cox proportional hazard models and adjusted for the first 10 principal components, sex and genotyping batch. CVD, cardiovascular disease; PGS, polygenic score for leisure screen time.

In FinnGen, those in the highest PGS LST decile had 19%–35% higher risk of CVDs compared with those in the lowest PGS LST decile (online supplemental table 5). For example, at 60 years of age, the cumulative incidence for any CVDs was 44% for those with high PGS LST, while for those with low PGS LST, it was 37% (online supplemental table 7, figure 3). Notably, both groups showed increasing trends, indicating that individuals with low PGS LST were also at risk of incident CVD (figure 3). This higher risk of incident CVD in participants with higher genetic liability replicated in HUNT, except for cerebrovascular disease (HR: 1.08 (95% CI 0.96 to 1.20)) (online supplemental table 6, online supplemental figure 1).

Considering other CVDs as competing events attenuated the cumulative incidence estimates for both high and low PGS LST participants, but their difference remained statistically significant for hypertensive diseases and ischaemic heart diseases, although not for cerebrovascular diseases (online supplemental table 8). In further sensitivity analyses with follow-up starting from individual DNA sampling dates (mean follow-up was 6.0 years in FinnGen and 5.0 in HUNT), the associations remained the same or were slightly attenuated in FinnGen and replicated in HUNT except for any CVD and cerebrovascular diseases (online supplemental table 9).

Exploratory analyses showed that the association between PGS LST and incident CVD persisted after adjustments for SES, BMI and smoking status, except for cerebrovascular diseases, where the number of cases was smallest (online supplemental figure 2). Adding the PGSs for CVDs and PGS PA attenuated the observed associations, indicating at least partial pleiotropy between CVD and sedentary behaviour, but not excluding other pathways for the observed effects (online supplemental table 10, online supplemental figure 3). Mediation analysis showed that higher PGS LST was associated with higher self-reported total sitting time (hour/day) in the HUNT cohort (β=0.06, SE=0.02, p=0.016 in a model for hypertensive diseases and β=0.07, SE=0.02, p=0.003 for ischaemic heart diseases) but indicated no potential mediating pathway to incident CVD (online supplemental figure 4). However, higher PGS LST was also associated with lower MET hour/week (β=−0.54, SE=0.09, p<0.001 for hypertensive diseases and β=−0.47, SE=0.09, p<0.001 for ischaemic heart diseases) and higher MET hour/week with lower risk of hypertensive and ischaemic heart diseases (β=−0.01, SE=0.00, p<0.001 and β=−0.01, SE=0.00, p=0.005, respectively). This suggests a potential mediating pathway from genetic liability to sedentary behaviour to incident CVD via insufficient levels of PA.

## Discussion

We found that higher genetic liability to LST was associated with more self-reported LST and that the genetic liability to LST also increased the risk of incident CVD, with replicable findings across two independent large populations. The observed effect sizes remained relatively small and attenuated after adjustment, but suggest that the observed adverse health effects may be either directly attributable to genetic effects or mediated by reduced levels of PA.

The underlying role of genetics in sedentary behaviour and morbidity has so far been understudied.^8^ Evolution has a profound influence on human biology; hence, evolution-based theories provide valuable insights into epidemiology and population health. Evolutionary selection processes may explain why the ‘desire to be sedentary is in our genes’ and also why previously advantageous alleles may currently be disease-causing alleles.^9 10 31^ In our study, the risk of any CVD increased very consistently by 5% per SD increase in the PGS LST. This magnitude is similar to what has been observed between every hour spent sedentary and risk of fatal and non-fatal CVD (relative risk=1.05, 95% CI=1.02 to 1.07).^32^ Furthermore, the risk of CVD differed significantly between those in the highest and lowest deciles of PGS LST. Those with high PGS LST had 21% (19% to 24%) higher risk for any CVD than those with low PGS LST, and at the age of 60, 7% higher cumulative CVD incidence. One explanation for these findings may be genetic pleiotropy.

In genetic pleiotropy, the same genes would affect both LST and CVD. This theory is supported by previously published genetic correlations between LST and several CVD-related phenotypes (0.24 for coronary artery disease, 0.32 for overweight, 0.22 for type 2 diabetes, −0.26 for high-density lipoprotein cholesterol and 0.27 for triglycerides), indicating low to moderate common genetic basis between LST and CVDs.^15^ Previous studies suggest that most robust pleiotropic genes are associated with the function of the nervous system, for example, the DLG4 gene (ENSG00000132535) is associated with synaptic function in the brain, pituitary gland and retina.^33 34^ Overall, the tissue enrichment analyses suggest that currently known genes associated with LST are expressed mainly in the nervous system, specifically in the brain.^15^ However, more studies are needed to further elucidate the underlying mechanism between genetic predisposition to LST and the onset of CVDs.

The observed coefficient of determination and effect size between PGS LST and self-reported LST were small, thus potentially raising concerns about how well the score reflects genetic liability to this trait. As there is little prior evidence on how PGSs are associated with sedentary behaviour while some data are available for PA, we discuss our findings aligned to this construct. Our results are consistent with previous studies in which PA PGSs were associated with the corresponding trait and differentiated well the extreme ends of the genetic spectrum, but explain only a small proportion (0.0%–1.4%) of the phenotypic variance.^21 35^ A plausible explanation for the small variances was assumed to be the heterogeneity in phenotype assessment (ie, PA collected through varying methods). However, in our study, we carefully selected the validation data to mimic the phenotype in the base data. Nevertheless, the fraction of variance explained remained small and may indicate other methodological limitations related to polygenic scoring. One of these is the ‘missing heritability’ dilemma. Missing heritability is the difference between the observed SNP heritability and heritability estimates based on twin studies (ranging from 19% to 56%).^36^,^39^ In our case, this is estimated to be at least 12% and potentially caused by the following: inflated family-based estimates,^40^ relatively small sample sizes of current GWASs,^41^ inadequate accuracy in phenotyping of the trait of interest and/or the lack of information on less common and rare variants in genotyping arrays used in GWAS studies. More detailed coverage of the variation in the genome by whole genome sequencing is expected to reduce missing heritability.^42 43^

### Policy implications

Our mediation analysis suggests that there may also be an alternative pathway in which sedentary genes contribute to inadequate levels of PA, thereby increasing the risk of incident CVD. Much has been written about the benefits of PA on cardiovascular health,^44^ with our findings bringing new perspectives to this discussion. Considering that genetics contribute to sedentary behaviour, effective public health strategies should emphasise approaches that nudge people to be active and/or interventions that include a social component and significantly nourish the intrinsic reward system.^45 46^ Previous work has suggested that systemic problems, like sedentariness, require systemic solutions.^46^ Whole population approaches to reduce sedentary time and/or increase PA are supported by our findings, where both high and low PGS LST groups showed increasing trends in CVD incidence. Moving from sedentary to active behaviour may confer health benefits, as the human evolutionary background may also explain why physiological functions operate optimally under regular PA.^47^ We acknowledge that sedentary behaviour may not fully displace PA behaviour,^48^ and the relationships between genetic liability to sedentary behaviour and observed behaviours throughout the day in different domains require further investigation.

### Strengths and limitations

This study has several strengths. We used high-performing and genome-wide methods for genetic scoring and showed the scores to be valid with independent data sets. We used the most comprehensive data available and a robust study design. We also acknowledge that our results should be interpreted with the following limitations in mind. The base data for PGS LST is based on self-reported LST which may be subject to biases from recall, cultural differences and social desirability.^49^ Future studies including larger sample sizes and device-based measurements of sedentary behaviour are warranted to construct better PGSs and to test the associations with CVDs. Notably, the LST represented screen time activities at the time of the data collection (2011) with limited generalisability to the modern era. The used FinnGen and HUNT may be enriched with CVDs due to participants having had an established contact with healthcare services. The FinnGen did not have data related to lifestyles, and therefore, the exploratory analyses could not be conducted in this cohort. Multiple testing may have increased the risk of Type I error, but it is controlled by the replication design.

## Conclusions

The findings of this study confirm our hypothesis that higher genetic liability for sedentary behaviour is associated with a greater risk of developing CVD, although effect sizes with the current data and methods remain small.

## Supplementary material

10.1136/bjsports-2024-109491 online supplemental file 1

10.1136/bjsports-2024-109491 online supplemental file 2

## Data Availability

Data are available on reasonable request. Data may be obtained from a third party and are not publicly available.

## References

[1] Kyu HH, Abate D, Abate KH (2018). Global, regional, and national disability-adjusted life-years (DALYs) for 359 diseases and injuries and healthy life expectancy (HALE) for 195 countries and territories, 1990-2017: a systematic analysis for the Global Burden of Disease Study 2017. Lancet.

[2] Tremblay MS, Aubert S, Barnes JD (2017). Sedentary Behavior Research Network (SBRN) - Terminology Consensus Project process and outcome. Int J Behav Nutr Phys Act.

[3] Husu P, Suni J, Vähä-Ypyä H (2016). Objectively measured sedentary behavior and physical activity in a sample of Finnish adults: a cross-sectional study. BMC Public Health.

[4] Yang L, Cao C, Kantor ED (2019). Trends in Sedentary Behavior Among the US Population, 2001-2016. JAMA.

[5] Biswas A, Oh PI, Faulkner GE (2015). Sedentary time and its association with risk for disease incidence, mortality, and hospitalization in adults: a systematic review and meta-analysis. Ann Intern Med.

[6] Ekelund U, Brown WJ, Steene-Johannessen J (2019). Do the associations of sedentary behaviour with cardiovascular disease mortality and cancer mortality differ by physical activity level? A systematic review and harmonised meta-analysis of data from 850 060 participants. Br J Sports Med.

[7] Raichlen DA, Lieberman DE (2022). The evolution of human step counts and its association with the risk of chronic disease. Curr Biol.

[8] Young DR, Hivert M-F, Alhassan S (2016). Sedentary Behavior and Cardiovascular Morbidity and Mortality: A Science Advisory From the American Heart Association. Circulation.

[9] Lea AJ, Clark AG, Dahl AW (2023). Evolutionary mismatch and the role of GxE interactions in human disease. ArXiv.

[10] Kirkwood TBL, Austad SN (2000). Why do we age?. Nature New Biol.

[11] Manus MB (2018). Evolutionary mismatch. Evol Med Public Health.

[12] Byars SG, Voskarides K (2020). Antagonistic Pleiotropy in Human Disease. J Mol Evol.

[13] Choi SW, Mak TS-H, O’Reilly PF (2020). Tutorial: a guide to performing polygenic risk score analyses. Nat Protoc.

[14] Piirtola M, Kaprio J, Ropponen A (2014). A study of sedentary behaviour in the older Finnish twin cohort: a cross sectional analysis. Biomed Res Int.

[15] Wang Z, Emmerich A, Pillon NJ (2022). Genome-wide association analyses of physical activity and sedentary behavior provide insights into underlying mechanisms and roles in disease prevention. Nat Genet.

[16] Bulik-Sullivan BK, Loh P-R, Finucane HK (2015). LD Score regression distinguishes confounding from polygenicity in genome-wide association studies. Nat Genet.

[17] Lloyd-Jones LR, Zeng J, Sidorenko J (2019). Improved polygenic prediction by Bayesian multiple regression on summary statistics. Nat Commun.

[18] Ni G, Zeng J, Revez JA (2021). A Comparison of Ten Polygenic Score Methods for Psychiatric Disorders Applied Across Multiple Cohorts. Biol Psychiatry.

[19] Herranen P, Palviainen T, Rantanen T (2022). A Polygenic Risk Score for Hand Grip Strength Predicts Muscle Strength and Proximal and Distal Functional Outcomes among Older Women. Med Sci Sports Exerc.

[20] Price AL, Patterson NJ, Plenge RM (2006). Principal components analysis corrects for stratification in genome-wide association studies. Nat Genet.

[21] Tynkkynen NP, Törmäkangas T, Palviainen T (2023). Associations of polygenic inheritance of physical activity with aerobic fitness, cardiometabolic risk factors and diseases: the HUNT study. Eur J Epidemiol.

[22] Joensuu L, Waller K, Kankaanpää A (2024). Genetic Liability to Cardiovascular Disease, Physical Activity, and Mortality: Findings from the Finnish Twin Cohort. Med Sci Sports Exerc.

[23] Tuomela J, Kaprio J, Sipilä PN (2019). Accuracy of self-reported anthropometric measures - Findings from the Finnish Twin Study. Obes Res Clin Pract.

[24] Therneau T survival: survival analysis. Version 3.5-8. https://cran.r-project.org/web/packages/survival/index.html.

[25] Alboukadel K, Marcin K, Przemyslaw B survminer: Drawing survival curves using “ggplot2”. Version 0.4.9. https://cran.r-project.org/web/packages/survminer/index.html.

[26] Vaura F, Kauko A, Suvila K (2021). Polygenic Risk Scores Predict Hypertension Onset and Cardiovascular Risk. Hypertension.

[27] Pace M, Cayotte E, Agafitei L (2013). Revision of the European standard population: report of Eurostat’s task force.

[28] Competing Risks Cumulative Incidence tidycmprsk 1.0.0. https://mskcc-epi-bio.github.io/tidycmprsk/reference/cuminc.html.

[29] VanderWeele TJ (2011). Causal mediation analysis with survival data. Epidemiology.

[30] Kieffer SK, Nauman J, Syverud K (2021). Association between Personal Activity Intelligence (PAI) and body weight in a population free from cardiovascular disease - The HUNT study. *Lancet Reg Health Eur*.

[31] Speakman JR (2020). An Evolutionary Perspective on Sedentary Behavior. Bioessays.

[32] Onagbiye S, Guddemi A, Baruwa OJ (2024). Association of sedentary time with risk of cardiovascular diseases and cardiovascular mortality: A systematic review and meta-analysis of prospective cohort studies. Prev Med.

[33] van de Vegte YJ, Said MA, Rienstra M (2020). Genome-wide association studies and Mendelian randomization analyses for leisure sedentary behaviours. Nat Commun.

[34] The human protein atlas: DLG4. https://www.proteinatlas.org/ENSG00000132535-DLG4.

[35] Kujala UM, Palviainen T, Pesonen P (2020). Polygenic Risk Scores and Physical Activity. Med Sci Sports Exerc.

[36] den Hoed M, Brage S, Zhao JH (2013). Heritability of objectively assessed daily physical activity and sedentary behavior. Am J Clin Nutr.

[37] van der Aa N, Bartels M, te Velde SJ (2012). Genetic and environmental influences on individual differences in sedentary behavior during adolescence: a twin-family study. Arch Pediatr Adolesc Med.

[38] Nelson MC, Gordon-Larsen P, North KE (2006). Body mass index gain, fast food, and physical activity: effects of shared environments over time. *Obesity (Silver Spring*).

[39] Schutte NM, Huppertz C, Doornweerd S (2020). Heritability of objectively assessed and self-reported sedentary behavior. Scand J Med Sci Sports.

[40] Muñoz M, Pong-Wong R, Canela-Xandri O (2016). Evaluating the contribution of genetics and familial shared environment to common disease using the UK Biobank. Nat Genet.

[41] Young AI (2019). Solving the missing heritability problem. PLoS Genet.

[42] Jang S-K, Evans L, Fialkowski A (2022). Rare genetic variants explain missing heritability in smoking. Nat Hum Behav.

[43] Wainschtein P, Jain D, Zheng Z (2022). Assessing the contribution of rare variants to complex trait heritability from whole-genome sequence data. Nat Genet.

[44] Wahid A, Manek N, Nichols M (2016). Quantifying the Association Between Physical Activity and Cardiovascular Disease and Diabetes: A Systematic Review and Meta-Analysis. J Am Heart Assoc.

[45] Lieberman DE (2015). Is Exercise Really Medicine? An Evolutionary Perspective. Curr Sports Med Rep.

[46] MacDonald C, Bennekou M, Midtgaard J (2025). Why exercise may never be effective medicine: an evolutionary perspective on the efficacy versus effectiveness of exercise in treating type 2 diabetes. Br J Sports Med.

[47] Eaton SB, Eaton SB (2003). An evolutionary perspective on human physical activity: implications for health. Comp Biochem Physiol A Mol Integr Physiol.

[48] Pearson N, Braithwaite RE, Biddle SJH (2014). Associations between sedentary behaviour and physical activity in children and adolescents: a meta-analysis. Obes Rev.

[49] Choi BCK, Pak AWP (2005). A catalog of biases in questionnaires. Prev Chronic Dis.

