# Supplementary material for: Genetic liability to sedentary behaviour and cardiovascular disease incidence in the FinnGen and HUNT cohorts
Source: Br J Sports Med. 2025 Mar 26;59(11):e109491. doi: 10.1136/bjsports-2024-109491 (PMC12171481; doi:10.1136/bjsports-2024-109491)
Supplement: online supplemental file 1 [file bjsports-59-11-s001.pdf]

June 2024 Volume 95 Issue 6

JOURNAL OF Neurology Neurosurgery & Psychiatry

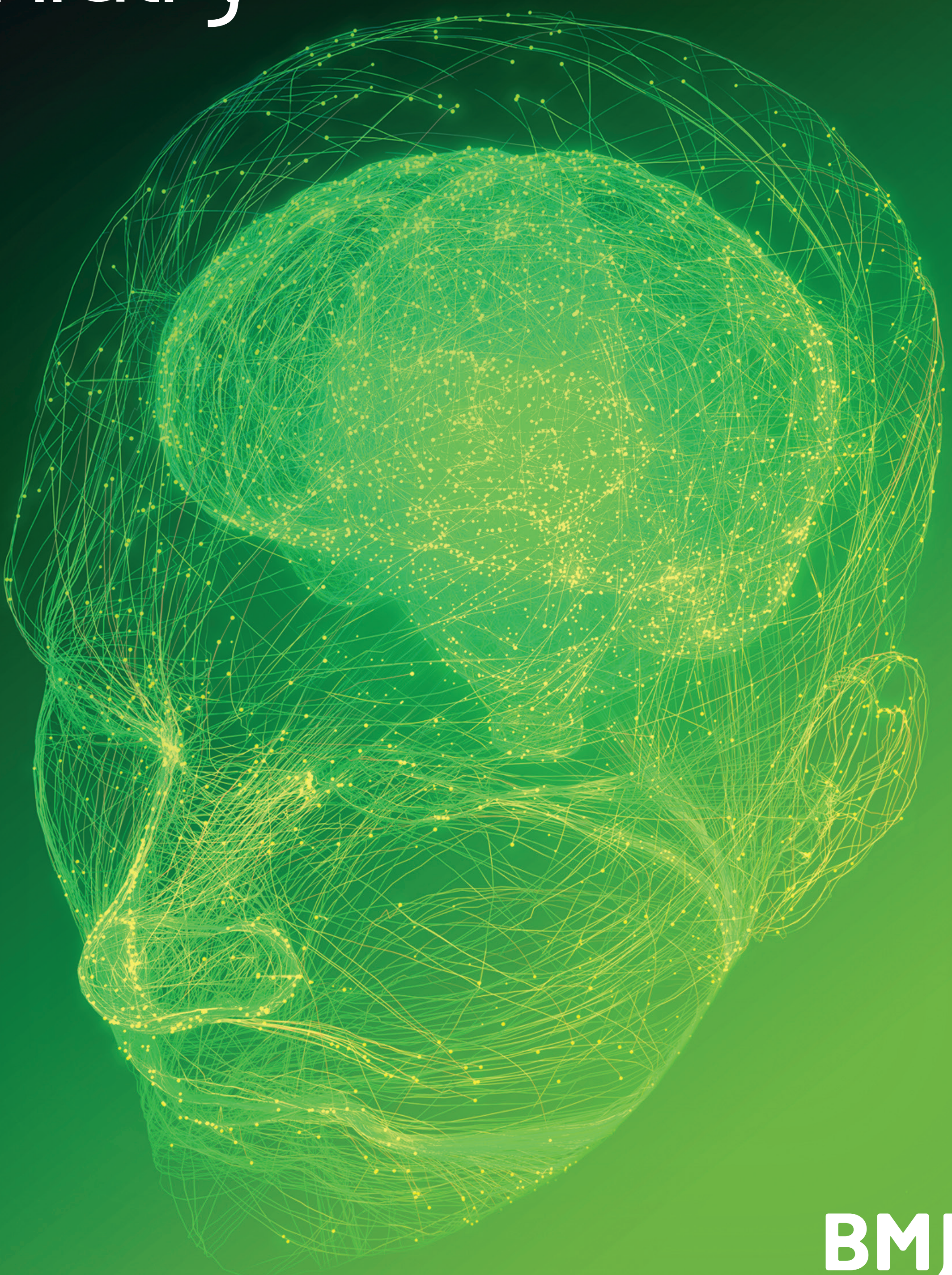

jnnp.bmj.com

BMJ
